# Preparation of a Cu(BTC)-rGO catalyst loaded on a Pt deposited Cu foam cathode to reduce CO_2_ in a photoelectrochemical cell

**DOI:** 10.1039/c8ra05964k

**Published:** 2018-09-18

**Authors:** Jun Cheng, Xiaoxu Xuan, Xiao Yang, Junhu Zhou, Kefa Cen

**Affiliations:** State Key Laboratory of Clean Energy Utilization, Zhejiang University Hangzhou 310027 China juncheng@zju.edu.cn +86 571 87951616 +86 571 87952889

## Abstract

To increase the reaction productivity and selectivity of the CO_2_ photoelectrochemical reduction reaction, a Cu (benzene 1,3,5-tricarboxylic acid [BTC])-reduced graphite oxide (rGO) catalyst was prepared by using a facile hydrothermal method and used in a CO_2_ photoelectrochemical cell (PEC) as a cathode catalyst. Characterization of the catalyst proved that successfully bonding of rGO to Cu(BTC) not only facilitated faster transfer of electrons on the surface of the catalyst but also created more active sites. CO_2_ photoelectrochemical reduction experimental results showed that the total carbon atom conversion rate was up to 3256 nmol h^−1^ cm^−2^ which was much higher than when pure Cu(BTC) was used as a cathode catalyst. The liquid product's selectivity to alcohols was up to 95% when −2 V voltage was applied to the system with Cu(BTC)-rGO used as the cathode catalyst.

## Abstract 2

Schematic of a photoelectrochemical cell for CO_2_ reduction: the H^+^ generation process and the CO_2_ process run in two separated chambers respectively.

## Graphical abstract

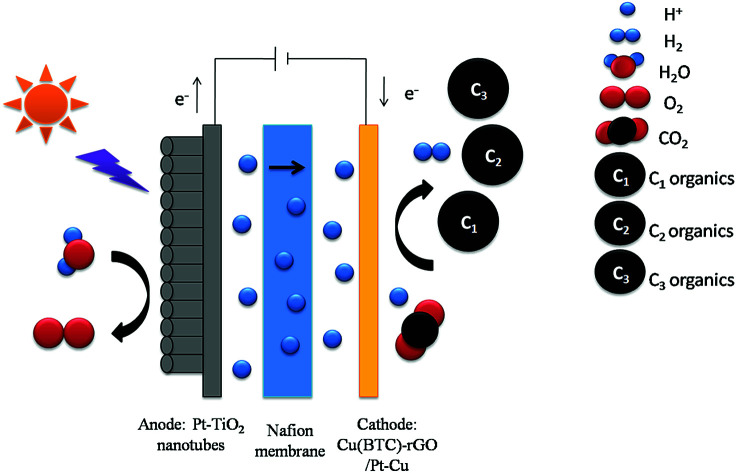

## 1. Introduction

Converting CO_2_ to clean fuels is considered an efficient way to solve both the problems of global warming and energy shortage at the same time.^1–4^ Artificial photosynthesis is a promising way in which CO_2_ is reduced to organic chemicals such as methanol, ethanol and formic acid, by using electricity and light with the help of catalysts.^5–7^

Given the large specific surface area and extremely high electron transition rate of graphene,^8,9^ using graphene as a CO_2_ reduction catalyst is a topic of great interest in the research field.^10^ Fan Yu Jia *et al.*^11^ studied the role that graphene played in the CO_2_ and H_2_ hydrocarbonylation reaction and graphene obviously helped increase the absorption of H_2_ and CO_2_, as well as the CO_2_ conversion rate. Moreover, selectivity toward methanol was increased to 85%. However, high pressure should be applied to ensure the proceeding of the reaction and this requires good resistance of the reactor. Geioushy R. A. *et al.*^12^ synthesized a Cu_2_O/graphene catalyst and used it in the CO_2_ electrochemical reduction reaction. The product analysis result showed that this catalyst had good selectivity toward ethanol generation. However, the energy used in this system totally came from electricity; renewable energies such as solar energy were not used. Cheng Jun *et al.*^13^ brushed the mixture of Nafion and reduced graphite oxide (rGO) on a Ni foam and used it as the cathode catalyst to reduce CO_2_ to valuable chemicals. However, the major products of Nafion and rGO are formic and acetic acids, which cannot be used as fuel directly. Moreover, the selectivity toward liquid fuels such as ethanol is low. Cu foam, which has 3D structure, is a good cathode base in electrochemical reactions. Cheng Jun *et al.*^14^ used Cu foam as a support base and loaded graphene on the matrix to reduce CO_2_ in a photoelectrochemical cell. However, the selectivity toward a specific product is still low.

Metal organic frameworks (MOFs), as an emerging porous material, have been reported in many researches.^15–21^ Numerous researchers have reported its outstanding performance in gas separation and catalytic reduction or oxidation.^22–25^ Bohan Shan *et al.*^26^ synthesized a novel Co-based MOF to separate CO_2_/N_2_ and CO_2_/CH_4_. Moreover, the CO_2_ separation rates have reached 61.4% and 11.7%, respectively. However, this Co-based MOF in the gas separation process is only a physical adsorption and desorption process but not in a chemical reaction. R. Senthil Kumar *et al.*^27^ used an electrochemical method, which successfully synthesized a highly active Cu-based MOF material and used it as a catalyst to reduce CO_2_ with an electrolyte consisting of a dimethylformamide solution of tetrabutyl ammonium tetra fluoroborate saturated with CO_2_. The result showed that this catalyst is an effective catalyst to reduce CO_2_ to organics. However, the organic synthesized was mainly oxalic acid and liquid fuels like ethanol and methanol were not obtained. Guoqiang Song *et al.*^28^ fabricated a porous Cu-benzene 1,3,5-tricarboxylic acid (BTC) silica monolith heterogeneous catalyst for the selective oxidation of alkylbenzenes. However, the usage of Cu(BTC) in CO_2_ photoelectrochemical reduction reactions has never been reported.

In this paper, a facile hydrothermal method was implied to synthesize Cu(BTC)-rGO catalyst, characterizations of the catalyst proved faster electron transfer rate and existence of more reaction active sites on the surface. These features facilitated the improvement of productivity and selectivity of CO_2_ photoelectrochemical reduction reaction.

## 2. Experiment

### 2.1. Cathode preparation

#### 2.1.1. GO synthesis

The GO used in this work was prepared using the Hummers' method.^29^ A total of 1 g high purity graphite was added into 25 ml of concentrated sulfuric acid. A total of 3.5 g of potassium permanganate was slowly added into the solution into the 0 °C ice bath; afterward, the mixed solution was stirred. The mixture was stirred and heated in an oil bath at a temperature of 35 °C for 2 h. Afterward, the mixture was diluted with 100 ml of deionized water and then oxidized with 8 ml of 30% H_2_O_2_ in the ice bath. The resulting suspension was washed with 5% diluted HCl, ultrasonically stripped, and was placed into a freeze dryer to be dried.

#### 2.1.2. Fabrication of Cu(BTC)-rGO/Pt–Cu foam cathode

Cu(BTC)-rGO was synthesized using a facile hydrothermal method. A total of 150 mg of as synthesized GO was dissolved in 15 ml of ethanol; this solution was marked as L1. A total of 0.93 g of Cu(NO_3_)_2_ was dissolved in 15 ml of DMF; this solution was marked as L2. A total of 0.44 g of BTC was dissolved in 30 ml of the ethanol and DMF mixture (volume, 1 : 1); this solution was marked as L3. Afterward, L1, L2, and L3 were mixed after stirring for 30 min. Then, the mixture was poured into a Teflon-lined autoclave after stirring for 30 min, and the autoclave was kept under 120 °C for 6 h. Black-blue precipitates were formed, centrifuged, and washed with methanol and deionized water several times to remove the undesirable impurities. Afterward, the precipitates were dried in dryer at 80 °C for 24 h. In this process, the added GO was transformed to rGO, given that GO was unstable under high temperature and can be easily reduced.

The Cu foam was deposited with Pt by using an electrochemical deposition method in a 0.1 g L^−1^ H_2_PtCl_4_ with an applied voltage of −0.2 V and deposition time of 180 s.

Then, the cathode was fabricated using the following method: 90 mg of as synthesized Cu(BTC)-rGO was mixed with the Nafion solution; afterward, the solution was placed on a Pt-deposited Cu foam using the drop–dry method.

#### 2.1.3. Fabrication of Cu(BTC)/Pt–Cu foam cathode

Blue mixture of Cu(BTC) and Nafion was obtained by mixing 90 mg Cu(BTC) with Nafion. Then, the mixture was applied on a Pt-deposited Cu foam using a drop–dry method.

### 2.2. Preparation of Pt–TiO_2_ nanotube anode

The Pt–TiO_2_ nanotubes (Pt-TNTs) were prepared based on the anode oxidation method. The titanium sheet was first rinsed in isopropanol and ethanol for 15 min for ultrasonic cleaning before oxidation. Afterward, the titanium sheet was oxidized in the fluorine-containing ethylene glycol electrolyte (EG + 0.3% NH_4_F + 2 vol% H_2_O) for 2 h, with 60 V applied voltage and Pt as the cathode. Then, the sheet was washed with deionized water and was placed into the muffle furnace to be calcined under 450 °C for 3 h, with the temperature rising rate of 2 °C min^−1^. Thus, the TiO_2_ nanotube crystals can be turned into anatase, which has better light response. The obtained anatase TiO_2_ nanotubes on the titanium sheet was deposited with Pt in 1 g L^−1^ of H_2_PtCl_6_·6H_2_O (depositing current density: 2.5 mA cm^−2^) to improve its photocatalytic performance.

### 2.3. CO_2_ photoelectrochemical reduction in a PEC

To test the performance of the as fabricated cathodes and find the best reaction condition of CO_2_ photoelectrochemical reduction. CO_2_ photoelectrochemical reduction reactions with two different cathodes were conducted, the reactions with different voltage applied on Cu(BTC)-rGO/Pd–Cu foam cathode were also conducted.

The CO_2_ photoelectrochemical reduction reaction was conducted in an H-shaped double chamber reactor. Then, a Nafion 117 membrane (Shanghai Hesen Co., Ltd.) was used to separate the two chambers. During the reaction process, light was applied to the anode Pt-TNT, and voltage was applied between the anode and cathode. The photocurrent was detected with CHI660D electrochemical workstation (CH instruments Co., Ltd.), and sunlight was simulated using Perfectlight PLS-SXE300CUV xenon lamp.

**Fig. 1:**
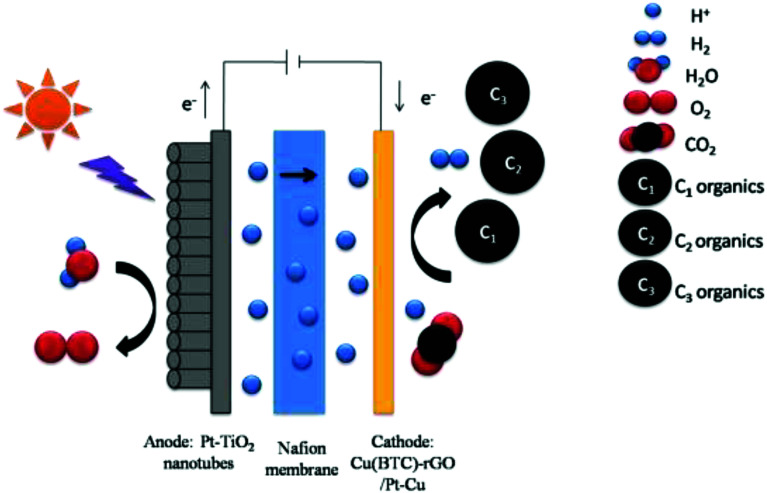
Schematic of photoelectrochemical cell for CO_2_ reduction.

The working area of cathode was 1 cm.^2^ The anolyte was 0.5 M H_2_SO_4_, while the catholyte was dimethylformamide (DMF). A water decomposition process happened on the anode Pt-TNT under light. The H^+^ generated by the oxidation of H_2_O under the catalytic effect of Pt-TNT crossed the Nafion membrane and took part in the CO_2_ reduction reaction on the cathode. At the same time, the photo-generated electrons went to the cathode through the external circuit. Afterward, CO_2_ and these electrons, as well as H^+^, reacted and produced organics under the catalytic effect of the cathode catalyst. The schematic of the photoelectrochemical cell for CO_2_ reduction is shown in Fig. 1. After the reaction, the liquid and gas products were collected and detected.

### 2.4. Characterization

The element analysis of Cu(BTC)-rGO and Cu(BTC) was conducted using a VG ESCALAB MARK II X-ray photoelectron spectroscopy (XPS) with Mg Kα radiation. X-ray diffraction (XRD) analysis was performed using an X'Pert PRO (PANalytical, Netherlands) diffractometer with Cu-Kα radiation. The morphologies of Cu(BTC)-rGO/Pt–Cu foam cathode and powder Cu(BTC)-rGO were characterized by field-emission scanning electron microscopy (SEM) using SU-8010 (Hitachi, Japan). The loading amount of Pt on the Cu foam was determined by the energy-dispersive X-ray spectroscopy (EDX) analysis on the SEM system equipped with an energy-dispersive X-ray analytical system. The Brunauer–Emmett–Teller (BET) analysis was conducted to measure the specific surface area and pore size of the as synthesized material on a Micromeritics ASAP 2020.

### 2.5. CO_2_ reduction product analysis

CO_2_ reduction products were analyzed offline. Liquid products, including alcohols and acids, were immediately sampled through the cell septum after the CO_2_ reduction reaction. Alcohols produced in the cell were analyzed using a gas chromatography system (GC; Agilent 7820A, USA) equipped with a thermal conductivity detector and a flame ionization detector. A column of DB-FFTP (*Φ*50 m × 0.32 mm × 0.5 mm) was used to detect alcohol. Acids were analyzed using an ion chromatography system (HPIC, Integrion, Dionex, USA) equipped with a conductivity detector and an AS11-HC-4 μm analytical column (4 × 250 mm).

## 3. Results and discussion

### 3.1. Cathode catalyst characterization

**Fig. 2:**
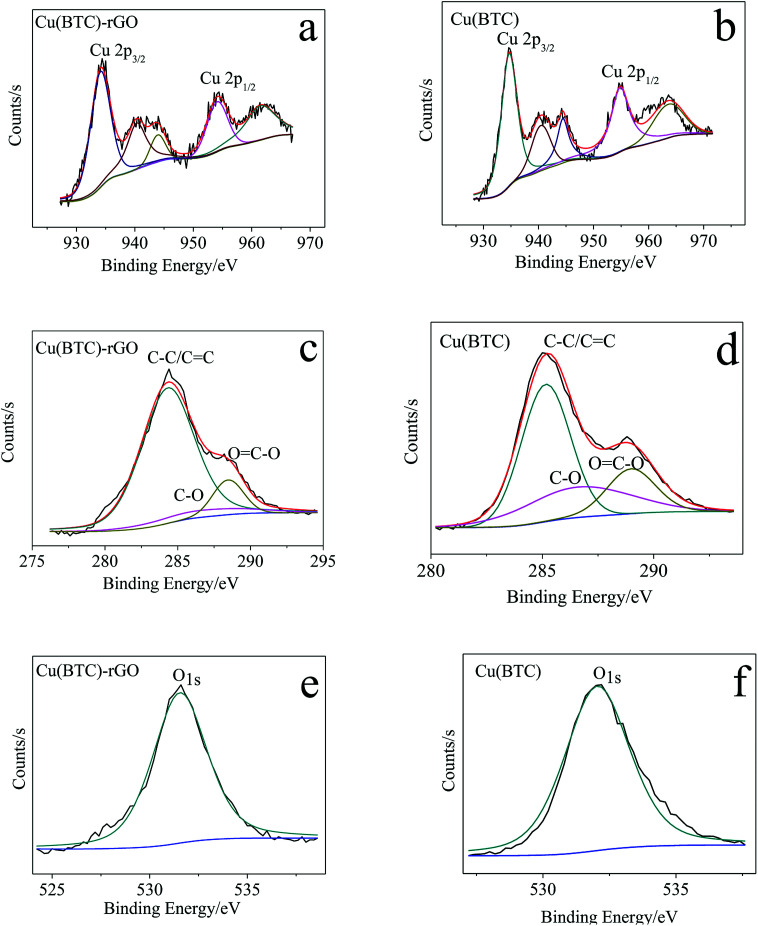
Cu 2p XPS spectra of Cu(BTC)-rGO (a) and Cu(BTC) (b); C 1s XPS spectra of Cu(BTC)-rGO (c) and Cu(BTC) (d); O 1s XPS spectra of Cu(BTC)-rGO (e) and Cu(BTC) (f).

To investigate the state of copper cluster and quantitatively analyze the element–atom ratio in the as obtained Cu(BTC)-rGO and Cu(BTC), we conducted XPS around the characteristic peak of Cu, C, and O. The XPS spectra of Cu, C, and O in Cu(BTC)-rGO and Cu(BTC) were shown in Fig. 2. Two characteristic peaks of divalent Cu^2+^ were observed at 934 eV and 954 eV, corresponding to Cu 2p^3/2^ and Cu 2p^1/2^, respectively. Meanwhile, the presence of the shake-up satellites, which are the other peaks that appeared in the range of 930–965 eV, except for two main characteristic peaks, found in the Cu spectra was an indication^30^ of the presence of Cu(ii) species in Cu(BTC)-rGO and Cu(BTC).

The quantitative analysis can be conducted by fitting the XPS spectra. The element atom number ratio in the samples was calculated by using the following equation:

1 *n*(*E*_1_)/*n*(*E*_2_) = [*A*(*E*_1_)/*S*(*E*_1_)]/[*A*(*E*_2_)/*S*(*E*_2_)]

Where *n* is the atom number, *E* is the element, *S* is the corresponding sensitive factor, and *A* is the area of characteristic peaks.

The calculation result showed that in Cu(BTC)-rGO, the element atom number ratios of Cu, C, and O were 4.9%, 23.7%, and 71.5%, respectively. Moreover, in Cu(BTC), the element atom ratios of Cu, C, and O were 5.7%, 18.4%, and 75.8%, respectively. The C atom ratio in Cu(BTC)-rGO was much higher than that in Cu(BTC). Further mining of the C 1s XPS spectra showed that three characteristic peaks were observed at 284 eV, 286 eV, and 289 eV, which correspond to C–C/C=C, C–O and O=C–O respectively. The weight ratios of these three functional groups were calculated. In Cu(BTC)-rGO, the ratios were 81.8%, 7.3%, and 10.9%, respectively. Moreover, in Cu(BTC), the ratios were 57.6%, 22.2%, and 20.2%, respectively. Thus, the successfully bonding of rGO in Cu(BTC)-rGO was proved; rGO bonding in the sample can accelerate the transfer of electron on the surface of the catalyst, leading to easier adsorption of the intermediates such as CO_2_* in the CO_2_ reduction reaction.^31,32^

**Fig. 3:**
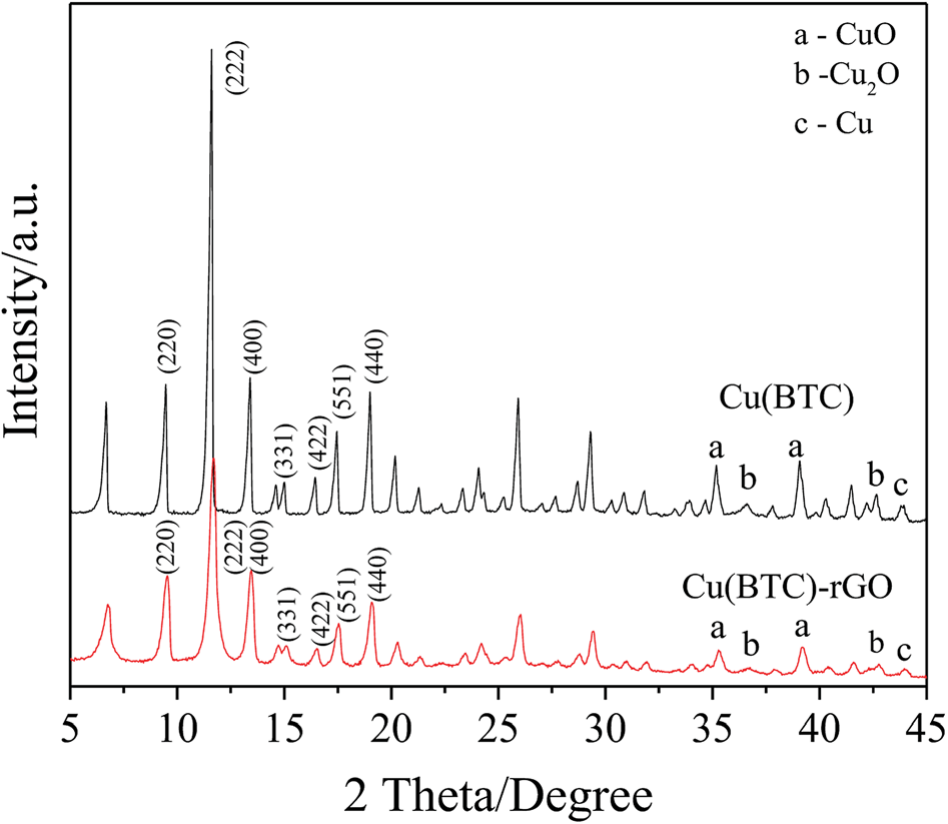
XRD patterns of Cu(BTC) and Cu(BTC)-rGO.

Successfully bonding of rGO into the Cu(BTC) crystal lattice in Cu(BTC)-rGO was also proven by the XRD pattern. As shown in Fig. 3, similar characteristic peaks in Cu(BTC)-rGO and Cu(BTC) were observed. However, the peaks in the Cu(BTC)-rGO XRD pattern shifted to the right due to rGO bonding. Both of the two samples consisted of a face-centered cubic crystal lattice of the *Fm*3̄*m* space group and had typical peaks at 2*θ* = 9.5° (220), 13.5° (400), 14.7° (331), 16.5° (422), 17.5° (511), and 19.1° (440). This result is in agreement with that of the previous reports.^33^ In addition to the characteristic peaks of the Cu(BTC) crystal, several small peaks in the range of 35°–45° were observed, which were the characteristic peaks of CuO, Cu_2_O, and Cu. This result indicated the synthesis of Cu and Cu_*x*_O in Cu(BTC)-rGO and Cu(BTC). According to a previous study,^12^ the mixture of Cu and Cu_*x*_O is an efficient catalyst to selectively reduce CO_2_ to ethanol; thus, the presence of these particle can promote the reaction selectivity to ethanol.

**Fig. 4:**
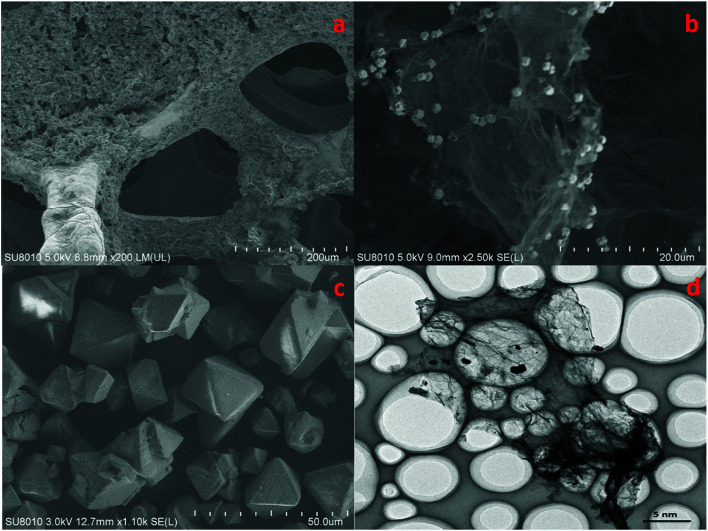
SEM patterns of Cu(BTC)-rGO/Pt–Cu foam cathode ×150 (a), Cu(BTC)-rGO catalyst ×2500 (b), Cu(BTC) catalyst ×1100 (c), TEM pattern of Cu(BTC)-rGO catalyst (d).

The morphology of cathode catalyst was shown in Fig. 4. Accordingly, Cu foam evidently had a 3D porous structure that can minimize the diffusion resistance for mass transport. This property of Cu foam favored the efficient transfer of CO_2_ and fast emission of products, which would accelerate the reaction rate.

Fig. 4(b) observed evidently wrinkled morphology, which was one of the typical characteristic of rGO. Many octahedral shaped Cu(BTC) particles were distributed uniformly on the rGO surface. Fig. 4(c) showed that morphology of Cu(BTC). TEM pattern of Cu(BTC)-rGO catalyst was given in Fig. 4(d), many octahedral shaped Cu(BTC) can also be observed bonded to the surfaced of rGO.

**Fig. 5:**
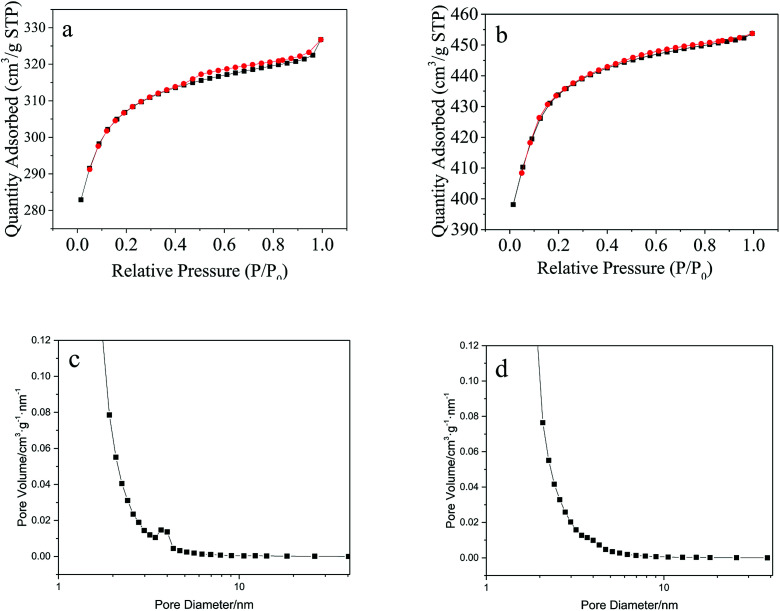
Nitrogen sorption isotherms: Cu(BTC)-rGO (a), Cu(BTC) (b); pore size distribution: Cu(BTC)-rGO (c), Cu(BTC) (d).

According to BET measurement results, the specific surface area of Cu(BTC)-rGO and Cu(BTC) were 938 m^2^ g^−1^ and 1322 m^2^ g^−1^ respectively. Though rGO bonding lead to the decrease of specific surface area of the catalyst, it successfully changed the pore structure of the catalysts. Fig. 5 showed the nitrogen sorption isotherms of Cu(BTC)-rGO (a) and Cu(BTC) (b). Accordingly, Cu(BTC)-rGO nitrogen adsorption plot showed a small hysteresis between adsorption and desorption branches suggesting the existence of few mesopores in the catalyst. However, Cu(BTC) nitrogen adsorption plot showed there only existed micropores in Cu(BTC). Pore size distribution [Fig. 5(c) and (d)] further confirmed this conclusion. The existence of mesopores better connected the micropores thus improving mass transfer rate.

Pt nanoparticles were supposed to be observed on the Cu foam. However, as the SEM pattern shows, Pt particles on the Cu foam matrix were hard to be observed mainly due to the small size. To identify the Pt content on the cathode, EDX analysis was conducted. EDX analysis showed that the Pt content on the cathode was about 0.56 wt%. The existence of Pt nanoparticles also contributed to facilitate charge transfer and intermediates adsorption on the surface of the catalyst.^34,35^

## 4. Cu(BTC)-rGO catalytic effect on the selectivity reduction of CO_2_ to ethanol

The CO_2_ photoelectrochemical reaction was performed in a novel photoelectrochemical reactor consisting of photo anode and electric cathode. A water decomposition process occurred on the anode Pt-TNT under light. Moreover, the generated H^+^ passed through the Nafion membrane and into the cathode. At the same time, the photo electrons went to the cathode through the external circuit, and CO_2_ and these electrons, as well as H^+^, reacted and produced organics under the catalytic effect of the cathode Cu(BTC)-rGO catalyst.

**Fig. 6:**
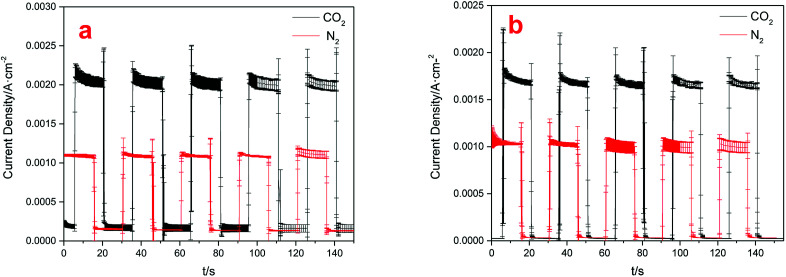
Electric current densities with different cathodes used: Cu(BTC)-rGO/Pt–Cu foam cathode (a), Cu(BTC)/Pt–Cu foam cathode (b).

Fig. 6 showed the current densities of the system when Cu(BTC)-rGO was used as catalyst (a), and Cu(BTC) was used as catalyst (b). The distance between TiO_2_ anode and light resource was 2 cm and the average incident light intensity provided at the electrode surface was measured to be 10 mW cm^−2^. Accordingly, the average current density under CO_2_ purging condition with Cu(BTC)-rGO used as catalyst was 2.13 mA cm^−2^. However, the average current density decreased to 1.75 mA cm^−2^ with Cu(BTC). This is a strong evidence to prove the improving of electron transfer rate on the surface when rGO is bonded to Cu(BTC). Moreover, when CO_2_ was bubbled into the solution, the current density showed sharp increase compared to that when N_2_ was bubbled.

**Fig. 7:**
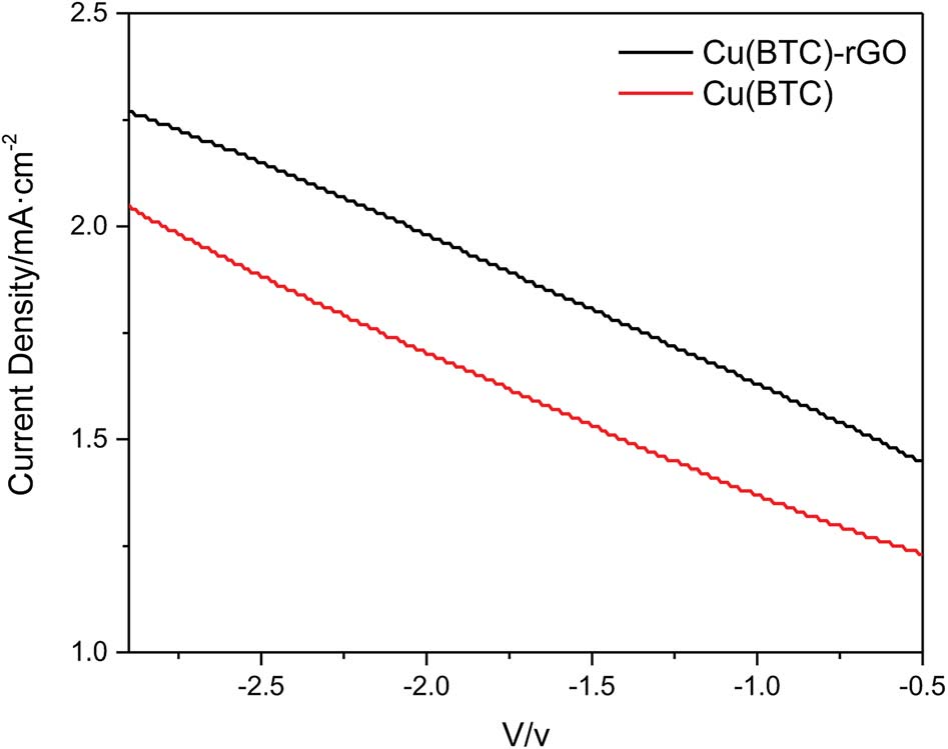
*J*–*V* curves of Cu(BTC)-rGO catalyst and Cu(BTC) catalyst obtained from CO_2_ electrochemical reduction in DMF (three electrode configuration, without photoanode).

The *J*–*V* curves of Cu(BTC)-rGO catalyst and Cu(BTC) catalyst obtained from CO_2_ electrochemical reduction in DMF (three electrode configuration, without photoanode) were shown in Fig. 7 and clearly, the current density of the system using Cu(BTC)-rGO catalyst was much higher than that when Cu(BTC) was used as catalyst.

**Fig. 8:**
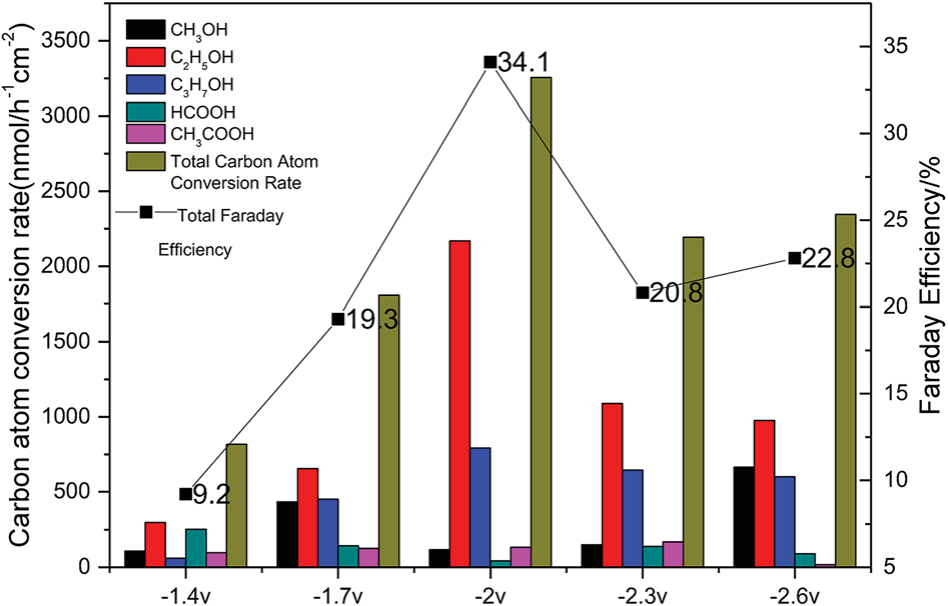
Carbon atom conversion rates and Faraday efficiencies with various applied voltage (−1.4 V, −1.7 V, −2 V, −2.3 V, −2.6 V) using Cu(BTC)-rGO/Pt–Cu foam cathode.

The results of the CO_2_ photoelectrochemical reduction experiment were shown in Fig. 8 Accordingly, the Cu(BTC)-rGO loaded on the Pt-deposited Cu foam cathode showed the best catalytic effect to reduce CO_2_ when the applied voltage on the system was −2 V. Moreover, the total carbon atom conversion rate reached 3255.87 nmol h^−1^ cm^−2^, and the liquid products selectivity towards alcohols was up to 95%. The Faraday efficiency was measured to be 34.1% with −2 V voltage applied.

The catalytic effect of Cu(BTC) under the condition with −2 V voltage applied was also studied. The result showed that the total carbon atom conversion rate was 2561.76 nmol h^−1^ cm^−2^, which was much lower than that when Cu(BTC)-rGO was used as the cathode catalyst. This result mainly due to the reason that lacking of rGO in the material will lead to lower transfer rate of electrons on the surface of the catalyst, resulting in the decrease of the carbon atom conversion rate.

## 5. Conclusion

In this research, Cu(BTC)-rGO was synthesized using a facile hydrothermal method. A Cu(BTC)-rGO/Pt–Cu foam cathode was fabricated and used to photoelectrochemically reduce CO_2_. The cathode showed the best catalytic effect with applied voltage of −2 V. The CO_2_ photoelectrochemical reduction result showed that the carbon atom conversion rate reached up to 3256 nmol h^−1^ cm^−2^, and the carbon atom conversion selectivity to liquid fuels was up to 94.6% when −2 V voltage was applied. The CO_2_ reduction mechanism on Cu(BTC)-rGO/Pt–Cu foam cathode was also proposed. In this process, rGO in the catalyst and the Pt nanoparticles on the Cu foam first transferred electron to CO_2_ to produce the C1 intermediates. As Pt showed good selectivity to the generation of CO, more CO than other C1 organics were produced. Then, the porous structure of Cu(BTC) facilitated C–C bonding, forming the enol-like intermediates that are key intermediates in alcohols generation. In conclusion, the bonding of rGO to Cu(BTC) is proved to be a promising way to improve the productivity and selectivity of CO_2_ photoelectreochemical reduction reaction. We believe that this would offer a new strategy for the synthesis of efficient CO_2_ photoelectrochemical reduction MOF based catalyst.

## Conflicts of interest

There are no conflicts to declare.

## Supplementary Material
